# MicroRNA‐Mediated Modulation of the Colony‐Stimulating Factor 1 Pathway in Microglial Activation and Neuropathic Pain: A Next‐Generation Sequencing‐Based Transcriptomic Study

**DOI:** 10.1155/prm/5465139

**Published:** 2026-08-30

**Authors:** Cheng-Chieh Kuo, Radika Tan, Yu-Yu Li, Meng-Hsun Hsieh, Kuo-Chuan Hung, Ping-Heng Tan

**Affiliations:** ^1^ Department of Anesthesiology, Chi-Mei Medical Center, Tainan 701, Taiwan, chimei.org.tw; ^2^ Department of Neuroscience and Behavioral Biology, College of Arts and Sciences, Emory University, Atlanta 30322, Georgia, USA, emory.edu; ^3^ Department of Anesthesiology, Chi-Mei Medical Center, Chiali, Tainan 722, Taiwan, chimei.org.tw; ^4^ Department of Leisure and Sports Management, CTBC University of Technology, Tainan 744, Taiwan; ^5^ School of Medicine, National Sun Yat-Sen University, Kaohsiung 800, Taiwan, nsysu.edu.tw

**Keywords:** colony-stimulating factor 1, colony-stimulating factor 1 receptor, glycosaminoglycans, microglia, miRNA, neuropathic pain, next-generation sequencing

## Abstract

**Objective:**

Neuropathic pain typically develops after nerve injury and is largely driven by microglial activation in the central nervous system (CNS), with colony‐stimulating factor 1 (CSF1) serving as a key initiator. We analyzed mRNA and microRNA expression profiles in CSF1‐stimulated primary microglia using next‐generation sequencing, followed by integrative bioinformatic analyses.

**Methods:**

Rat microglia were cultured in microglial medium at 37°C with 5% CO_2_ and treated with CSF1 (20 ng/mL) for 16 h to assess early transcriptional and miRNA–mRNA responses. Total RNA was extracted using TRIzol and quantified using a Nanodrop and Bioanalyzer. RNA libraries were prepared with the SureSelect kit and sequenced on an Illumina platform. Differentially expressed mRNAs were defined as those with a fold change ≥ 2 and an FDR‐adjusted *p* value < 0.05, whereas differentially expressed miRNAs were identified using a fold change ≥ 1.5 for both upregulated and downregulated miRNAs with a *p* value < 0.05. The miRNA–mRNA interactions were assessed using miRWalk, and KEGG/GO analyses identified pathways related to inflammation, immune signaling, and extracellular matrix (ECM) remodeling.

**Results:**

CSF1 stimulation altered the expression of 176 miRNAs and 429 mRNAs, including 86 upregulated and 90 downregulated miRNAs. Inverse correlation analysis revealed 68 downregulated miRNAs linked to 119 upregulated mRNAs, and 69 upregulated miRNAs linked to 204 downregulated mRNAs, indicating complex regulatory interactions. The transcriptional program induced by CSF1 was characterized by increased expression of proinflammatory and proliferative genes, such as Fos, Cxcl2, and Ephb3, and suppressed expression of ECM‐ and glycosaminoglycan (GAG)‐related genes, including Gpc6 and Prelp. Several miRNAs, including rno‐miR‐652‐5p, rno‐miR‐672‐5p, rno‐miR‐455‐3p, rno‐miR‐145‐5p, rno‐miR‐222‐3p, rno‐miR‐702‐5p, rno‐miR‐877, rno‐miR‐664‐2‐5p, and rno‐miR‐702‐3p, emerged as potential upstream regulators of these changes. Pathway enrichment analysis highlighted the activation of TNF, IL‐17, and mitogen‐activated protein kinase (MAPK) signaling, coupled with the suppression of ECM remodeling.

**Conclusion:**

These findings identify candidate miRNA–mRNA regulatory networks associated with CSF1‐driven microglial responses and provide new insights into transcriptomic changes relevant to neuroimmune mechanisms in neuropathic pain.

**Summary**
• This study identifies miRNA–mRNA regulatory networks underlying CSF1‐induced microglial activation, revealing proinflammatory and ECM‐suppressive transcriptional programs that may contribute to the pathogenesis of neuropathic pain.

## 1. Introduction

Neuropathic pain is a chronic, debilitating condition resulting from injury to or dysfunction of the nervous system. Neuropathic pain affects approximately 7%–10% of the global population and remains a major clinical challenge because it does not respond to standard analgesics; moreover, it significantly affects the quality of life of patients [1, 2]. There is a growing recognition that central neuroimmune interactions, particularly those involving microglial activation, play a significant role in the development and maintenance of neuropathic pain [3, 4]. Following peripheral nerve injury, resident central nervous system (CNS) immune cells and microglia undergo rapid morphological and functional changes to increase pain sensitivity; however, the molecular regulators that orchestrate these responses are not fully understood, consistent with broader evidence indicating that microglia are central yet heterogeneous players across diverse brain diseases [5].

Colony‐stimulating factor 1 (CSF1), a cytokine important for microglial proliferation, survival, and functional reprogramming after nerve injury, is among the mediators of microglial activation [6, 7]. CSF1 signaling through its receptor, CSF1R, induces transcriptional modifications that facilitate a shift from homeostatic to reactive microglial phenotypes, including increased expression of several genes such as Dap12 [8], Irf8/5 [9, 10], P2rx4 [10, 11], Cx3cr1 [12–14], the proinflammatory cytokines TNF and IL‐1β, and the neurotrophins BDNF [15] and CTSS [16]. These findings establish CSF1–CSF1R signaling as a central pathway coupling microglial transcriptional reprogramming with neuroimmune interactions that promote pain sensitization [6, 17, 18]. While CSF1 is significantly involved in microglial remodeling and pain sensitization in preclinical models [7, 19], the upstream regulatory mechanisms that regulate CSF1‐mediated transcriptional responses have not been thoroughly investigated.

MicroRNAs (miRNAs), a class of small noncoding RNAs that posttranscriptionally control gene expression, have recently been recognized as important regulators of the modulation of neuroimmune responses [20]. Because miRNAs simultaneously target several genes within signaling cascades, they are well positioned to coordinate complex cellular responses. In microglia, miRNAs regulate intracellular signaling pathways that control essential functions, such as cytokine production, phagocytic activity, and phenotypic transitions between homeostatic and reactive states in response to environmental stimuli [21–23]. Changes in the expression of several miRNAs contribute to the development of neuropathic pain through the modulation of neuroinflammatory signaling networks, the regulation of ion channel expression, and increased neuronal excitability, all of which lead to the establishment of pain hypersensitivity and are therefore attractive diagnostic and therapeutic targets in preclinical and clinical settings [22–24]. Furthermore, altered miRNA expression profiles have been observed in a wide range of neurological disorders, such as Alzheimer’s disease and multiple sclerosis, where they are thought to contribute to disease progression by disrupting immune homeostasis, glial activation, and neuronal integrity [24, 25]. However, whether and how miRNAs modulate CSF1‐induced microglial activation are not well defined. Thus, in the present study, we analyzed the expression profiles of miRNAs and genes and performed integrated bioinformatics analyses to identify specific miRNA–mRNA regulatory networks in CSF1‐stimulated microglia in vitro.

## 2. Materials and Methods

### 2.1. Cell Culture and CSF1 Treatment

Rat microglia (RMs) were obtained from ScienCell Research Laboratories (Carlsbad, California, USA) and were isolated from postnatal Day 2 rat brains and cultured under standard sterile conditions in microglial medium (MM; Cat. #1901) for in vitro culture. The microglia were maintained in humidified air containing 5% CO_2_ at 37°C. Primary microglia were stimulated with CSF1 (20 ng/mL, PeproTech) for 16 h under standard culture conditions (37°C and 5% CO_2_), and the vehicle control microglia were treated with PBS. This time window was selected to capture early transcriptional reprogramming and miRNA–mRNA interactions in response to CSF1, which is consistent with previous studies, showing that 12–24 h of stimulation effectively reveals microglial activation signatures without confounding long‐term culture effects [26].

### 2.2. RNA Extraction

Total RNA was extracted using TRIzol Reagent (Invitrogen, USA) according to the manufacturer’s instructions. Purified RNA was quantified at OD260 nm using an ND‐1000 spectrophotometer (Nanodrop Technology, USA), and the RNA concentration was quantified via a Bioanalyzer 2100 (Agilent Technology, USA) with an RNA 6000 LabChip kit (Agilent Technology, USA).

### 2.3. Next‐Generation Sequencing (NGS)

Total RNA samples from three independent biological replicates per group (CSF1‐treated microglia and vehicle controls) were subjected to NGS. RNA integrity was assessed using an Agilent Bioanalyzer 2100, and samples with an RNA integrity number (RIN) ≥ 7 were used for library preparation.

RNA‐seq libraries were generated using the SureSelect Strand‐Specific RNA Library Preparation Kit (Agilent Technologies, USA) according to the manufacturer’s protocol. Libraries were purified and size‐selected using AMPure XP beads (Beckman Coulter, USA). Sequencing was performed on an Illumina sequencing platform using sequencing‐by‐synthesis (SBS) chemistry. On average, ∼25–30 million paired‐end reads per sample were generated. Raw sequencing reads were processed through Welgene Biotech’s standard analysis pipeline, and base calling was performed using Illumina bcl2fastq v2.20 to generate FASTQ files for downstream analysis.

#### 2.3.1. miRNA Library Preparation and Quantification

Small RNA libraries were prepared and sequenced by Welgene Biotech (Taipei, Taiwan) using the QIAseq miRNA Library Kit (Qiagen, Hilden, Germany) according to the manufacturer’s instructions. Libraries were sequenced on the Illumina NextSeq 500 platform.

Raw BCL files were converted to FASTQ format using Illumina bcl2fastq Conversion Software v2.20. Adapter sequences and low‐quality bases were removed using Trimmomatic v0.36. Qualified reads were aligned to the *Rattus norvegicus* reference genome using miRDeep2, which was employed to quantify known miRNAs and predict novel miRNAs. Only reads mapping perfectly to the genome at no more than five genomic locations were retained for downstream analysis. Known miRNAs were annotated according to miRBase release 21. miRNA expression levels were normalized as reads per million mapped reads (RPM), and differential expression analysis was performed using the Welgene Biotech bioinformatics pipeline.

### 2.4. Differential Expression and Statistical Analysis

Raw sequencing reads were quality‐checked and aligned to the rat reference genome using the Welgene Biotech bioinformatics pipeline. Gene expression levels were quantified as fragments per kilobase of transcript per million mapped reads (FPKM), and differential gene expression analysis between CSF1‐treated and control microglia was performed using Cuffdiff (Cufflinks v2.2.1) with genome bias correction enabled. Transcripts with very low abundance (FPKM < 0.3 in either condition) were excluded to reduce noise. Statistical significance of differential expression was determined using the Benjamini–Hochberg false discovery rate (FDR) correction to control for multiple hypothesis testing. Differentially expressed mRNAs were defined as those with fold change ≥ 2 and an FDR‐adjusted *p* value < 0.05. Differentially expressed miRNAs were identified using a fold change ≥ 1.5 for both upregulated and downregulated miRNAs, with a *p* value < 0.05. A lower fold change was applied to miRNAs because miRNAs often exhibit more modest expression changes than mRNAs while still exerting biologically meaningful regulatory effects. This approach was adopted to retain candidate regulatory miRNAs for downstream miRNA–mRNA integration analysis. For miRNA differential expression analysis, a nominal (uncorrected) *p* value < 0.05 in combination with a fold‐change threshold was used instead of FDR adjustment because the miRNA dataset contained substantially fewer features than the mRNA dataset, and the analysis was intended as an exploratory screening step to identify candidate regulatory miRNAs for subsequent experimental validation. Therefore, priority was given to minimizing false negatives rather than applying a more stringent multiple‐testing correction at this discovery stage.

Functional enrichment analyses were performed using clusterProfiler (v3.6) for Gene Ontology (GO) and Kyoto Encyclopedia of Genes and Genomes (KEGG) pathway analysis.

### 2.5. miRNA–mRNA Integration Analysis

To identify potential regulatory interactions between miRNAs and their target mRNAs, inverse expression matching was performed between differentially expressed miRNAs and mRNAs. Target prediction was conducted using the miRWalk 3.0 database, which integrates multiple prediction algorithms. Only miRNA–mRNA pairs showing inverse expression patterns (upregulated miRNAs with downregulated target mRNAs, or downregulated miRNAs with upregulated target mRNAs) were retained for further analysis.

For functional enrichment analysis, the integrated miRNA–mRNA pairs were ranked according to mRNA expression levels. The top 50 most highly expressed genes among the upregulated mRNAs and the bottom 60 lowest expressed genes among the downregulated mRNAs were selected for GO and KEGG pathway enrichment analyses.

### 2.6. Pathway and Functional Enrichment Analysis

The selected mRNAs derived from the miRNA–mRNA integration analysis were subjected to GO and KEGG pathway enrichment analysis using clusterProfiler. Statistical significance for enrichment analyses was determined using FDR‐adjusted *p* values, with FDR ≤ 0.05 considered statistically significant. Biological pathways associated with inflammation, immune signaling, microglial activation, and extracellular matrix (ECM) remodeling were prioritized for interpretation. Based on these results, key regulatory miRNAs potentially involved in CSF1‐induced microglial activation were identified for further investigation.

## 3. Results

### 3.1. Differential Expression of miRNAs and mRNAs

**FIGURE 1:**
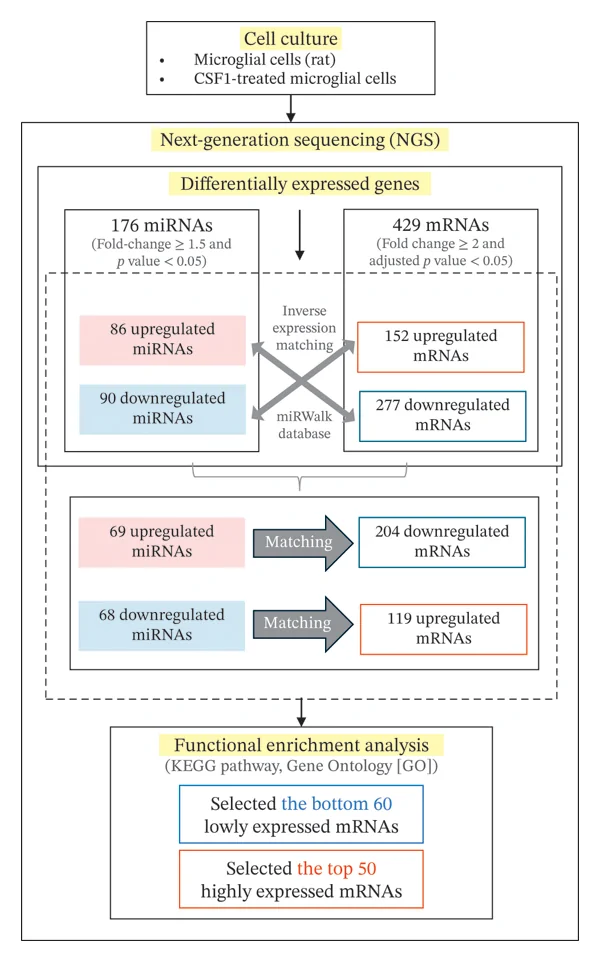
Overview of experimental flowchart.

**FIGURE 2:**
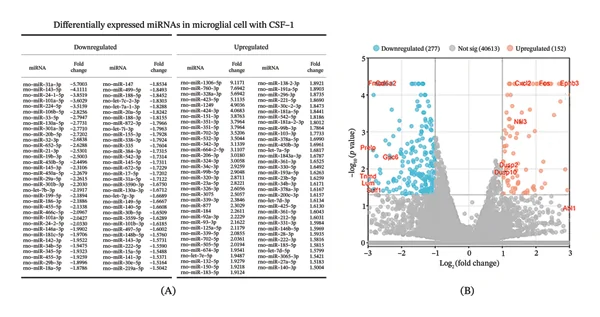
Differential expression of miRNAs and mRNAs in CSF1‐stimulated microglia. (A) Differentially expressed miRNAs identified by next‐generation sequencing (NGS). A total of 176 differentially expressed miRNAs, including 86 upregulated and 90 downregulated miRNAs, were identified using a fold change ≥ 1.5 for both upregulated and downregulated miRNAs (*p* < 0.05). (B) Volcano plot showing the differential expression of 41,042 mRNAs. Significantly upregulated mRNAs are shown in red (*n* = 152), while downregulated mRNAs are shown in blue (*n* = 277). Gray points indicate mRNAs with no significant differential expression. Differentially expressed mRNAs were identified using a fold change ≥ 2 and an FDR‐adjusted *p* value < 0.05. A few of the genes are related to the inflammatory response, such as Fos, Cxcl2, and Prelp, whereas Gpc6 is associated with the extracellular matrix.

The flowchart of the present study is illustrated in Figure 1. To examine the transcriptomic effects of CSF1 on activated microglia, RNA was extracted from microglia that had been treated with recombinant CSF1 or a vehicle control for 16 h. The extracted RNA samples were subjected to NGS to evaluate both mRNA and miRNA expression profiles. A total of 176 differentially expressed miRNAs were identified, consisting of 86 upregulated and 90 downregulated miRNAs, based on the criteria of fold change ≥ 1.5 in either the upregulated or downregulated direction and *p* value < 0.05 Figure 2A. These miRNAs are potentially involved in regulating signaling cascades associated with microglial activation and inflammation. Differential expression analysis of 41,042 mRNAs was performed, and the results were visualized using a volcano plot to show the distribution of expression changes Figure 2B. In total, 429 mRNAs were significantly differentially expressed, including 152 upregulated and 277 downregulated mRNAs, according to the predefined criteria of a fold change ≥ 2 and an FDR‐adjusted *p* value < 0.05. Notably, several proinflammatory and proliferative genes, including Fos, Cxcl2, Dusp2, and Ephb3, were significantly upregulated following CSF1 exposure, which is consistent with a phenotype of microglial activation. In contrast, genes associated with ECM organization and glycosaminoglycan (GAG) metabolism, such as Gpc6, Prelp, Col6a2, and Lum, were markedly downregulated. Collectively, these data indicate that CSF1 stimulation promotes an inflammatory and proliferative transcriptional program in microglia while suppressing ECM‐associated homeostatic pathways.

### 3.2. Inverse Expression Matching and Pathway Enrichment Analysis

**FIGURE 3:**
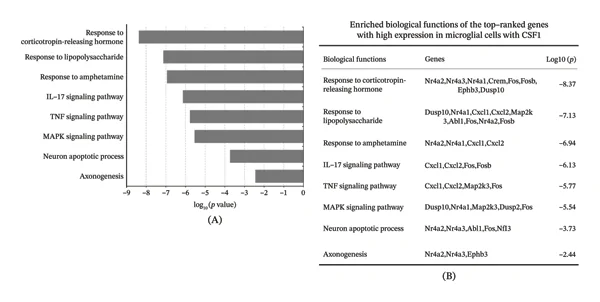
Biological functions of the top 50 most highly expressed genes among the upregulated mRNAs following CSF1 stimulation. (A) Bar chart of the enriched biological functions for the top 50 most highly expressed genes among the upregulated mRNAs in CSF1‐treated microglia. The *x*‐axis represents the log_10_ (*p* value), which indicates the significance of enrichment. Biological function annotation was performed using the KEGG and Gene Ontology (GO) databases. (B) Genes related to biological functions and signaling pathways identified by KEGG and GO enrichment analyses. The genes listed are among the top 50 most highly expressed genes within the upregulated mRNAs in CSF1‐stimulated microglia.

To investigate the regulatory interactions between miRNAs and mRNAs in CSF1‐stimulated microglia, an inverse expression matching analysis was conducted. Among the 176 differentially expressed miRNAs identified through NGS (86 upregulated and 90 downregulated), 68 downregulated miRNAs were inversely matched with 119 upregulated mRNAs. Concurrently, 69 upregulated miRNAs were inversely matched with 204 downregulated mRNAs. For functional enrichment analysis, the integrated mRNA candidates were ranked according to their expression levels. The top 50 most highly expressed genes among the upregulated mRNAs and the 60 lowest expressed genes among the downregulated mRNAs were selected for GO and KEGG pathway enrichment analyses. The biological functions in which the 50 most highly expressed upregulated genes were enriched following CSF1 stimulation are illustrated in Figure 3A. Among the most significantly enriched terms identified were the response to corticotropin‐releasing hormone (log_10_ (*p* value) = −8.37), response to lipopolysaccharide (log_10_ (*p* value) = −7.13), and response to amphetamine (log_10_ (*p* value) = −6.94). These findings suggest increased reactivity of CSF1‐stimulated microglia to neuroendocrine and inflammatory stimuli. Such enrichment implies transcriptional priming of microglia in response to stress, immune, and neurotransmitter signals. Additionally, significant enrichment of genes related to IL‐17 signaling (log_10_ (*p* value) = −6.13), TNF signaling (log_10_ (*p* value) = −5.77), and mitogen‐activated protein kinase (MAPK) signaling (log_10_ (*p* value) = −5.54) was detected. These findings indicate that activated microglia participate in cytokine‐mediated inflammatory cascades, which are characteristics of neuroinflammation. Moreover, genes associated with neurodevelopment and cell death processes, such as the neuron apoptotic process (log_10_ (*p* value) = −3.73) and axonogenesis (log_10_ (*p* value) = −2.44), were also enriched. The genes associated with biological functions and signaling pathways identified through KEGG and GO analyses are shown in Figure 3B.

Integration of the miRNA–mRNA interaction data (Table 1) revealed that several upregulated genes were predicted targets by multiple downregulated miRNAs, including rno‐miR‐652‐5p, rno‐miR‐672‐5p, rno‐miR‐455‐3p, and rno‐miR‐145‐5p. Among the predicted target genes, Ephb3, Nr4a2, and Nr4a1 were consistently identified across multiple miRNA regulatory networks. Given their established roles in transcriptional regulation and cellular proliferation, these genes may represent important components of the microglial response to CSF1 stimulation.

**TABLE 1:** Upstream microRNAs targeting top‐ranked upregulated genes in CSF1‐treated microglial cells.

microRNA	Rno‐miR‐652‐5p	Rno‐miR‐672‐5p	Rno‐miR‐455‐3p	Rno‐miR‐145‐5p
Genes	Abl1	Abl1	Crem	Abl1
	Crem	Crem	Cxcl2	Dusp10
	Dusp10	Cxcl2	^#^Ephb3	^#^Nr4a1
	^#^Ephb3	Dusp10	Map2k3	^#^Nr4a2
	Fos	^#^Nr4a2	Nfil3	
	Fosb			

^#^Cell proliferation‐related genes.

Notably, Nr4a1 and Nr4a2 are immediate‐early response genes induced by inflammatory signaling and have been implicated in regulating microglial activation and inflammatory responses. Increasing evidence suggests that NR4A family members function as counter‐regulatory transcription factors that help limit excessive inflammatory signaling in activated microglia. Therefore, the coordinated upregulation of Nr4a1/Nr4a2 and downregulation of their predicted regulatory miRNAs observed in the present study may reflect a compensatory transcriptional response to CSF1 stimulation. Because the miRNA–mRNA interactions identified in this study were based on bioinformatic prediction, these regulatory relationships require further experimental validation. Nevertheless, the integrated analysis suggests that miRNA‐mediated regulatory networks may contribute to the transcriptional response of microglia following CSF1 stimulation.

Additionally, differential expression analysis of genes associated with microgliosis between the CSF1‐stimulated and vehicle control groups was conducted (Table 2). The graph illustrates relative expression levels, revealing a marked increase in the expression of key microgliosis‐related genes following CSF1 stimulation. This observation suggests that CSF1 plays a crucial role in fostering a reactive microglial phenotype, characterized by the upregulation of genes associated with proliferation, signaling response, and inflammation.

**TABLE 2:** Comparative expression analysis of microgliosis‐related genes in CSF1‐stimulated versus control microglial cells.

Genes	CSF/ctrl	*p* value
Ephb3	16.02	0.00005
Vegfa	8.58	0.00005
Nr4a2	6.45	0.00005
Nr4a3	4.81	0.00005
Cxcl1	2.51	0.0038
Pim 1	1.91	0.00875
Notch 1	1.77	0.00765

**FIGURE 4:**
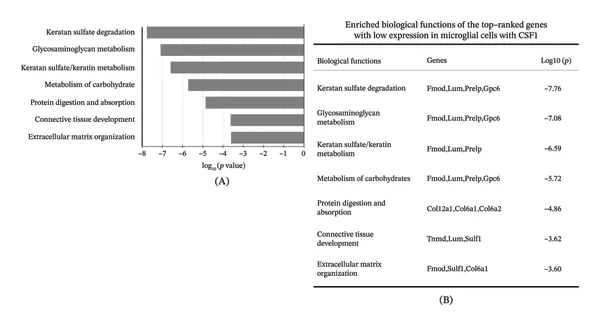
Biological functions of the bottom 60 lowest expressed genes among the downregulated mRNAs following CSF1 stimulation. (A) Bar chart showing the biological functions of the bottom 60 lowest expressed genes among the downregulated mRNAs in CSF1‐treated microglia. The *x*‐axis indicates the log_10_ (*p* value), which indicates the enrichment significance. Biological functions were annotated using the KEGG and Gene Ontology (GO) databases. (B) Genes associated with biological functions and signaling pathways according to KEGG and GO enrichment analyses. The genes were among the bottom 60 lowest expressed genes within the downregulated mRNAs in CSF1‐stimulated microglia.

To clarify the functional consequences of the downregulated genes following CSF1 treatment, we conducted pathway enrichment analysis of the bottom 60 lowest expressed genes among the downregulated mRNAs, which revealed statistically significant enrichment of several biological processes and pathways Figure 4. Among these, the most prominently enriched processes and pathways were linked to keratan sulfate degradation (log_10_ (*p* value) = −7.76), GAG metabolism (log_10_ (*p* value) = −7.08), and keratan sulfate/keratin metabolism (log_10_ (*p* value) = −6.59), suggesting potential disruption of ECM remodeling. Furthermore, additional pathways, such as carbohydrate metabolism, protein digestion and absorption, connective tissue development, and ECM organization, were also significantly affected. These results imply that CSF1 stimulation may inhibit genes that are crucial for structural tissue maintenance and metabolic functions, aligning with the notion that microglial activation can lead to ECM reorganization and alteration of nutrient processing. Further analysis of the upstream regulatory miRNAs targeting these 60 lowest expressed mRNAs (Table 3) revealed rno‐miR‐222‐3p, rno‐miR‐702‐5p, rno‐miR‐877, rno‐miR‐664‐2‐5p, and rno‐miR‐702‐3p as potential regulators. These miRNAs commonly target several genes, such as Gpc6, Prelp, and Fmod, all of which are functionally associated with GAG metabolism. These findings suggest a potential miRNA‐mediated regulatory mechanism in the suppression of ECM remodeling in CSF1‐treated microglia.

**TABLE 3:** Upstream microRNAs targeting top‐ranked downregulated genes in CSF1‐treated microglial cells.

microRNA	Rno‐miR‐222‐3p	Rno‐miR‐702‐5p	Rno‐miR‐877	Rno‐miR‐664‐2‐5p Rno‐miR‐702‐3p
Genes	Col6a1	Col6a1	Col12a1	Col6a2
	Col6a2	^∗^Fmod	Col6a2	^∗^Gpc6
	^∗^Gpc6	^∗^Gpc6	^∗^Fmod	
	^∗^Prelp	Tnmd		

^∗^Glycosaminoglycan metabolism‐related gene.

## 4. Discussion

In the present study, the transcriptomic landscape of CSF1‐stimulated microglia was elucidated, with the identification of key miRNAs that regulate inflammatory and ECM‐related pathways, thus providing novel information about the posttranscriptional mechanisms underlying neuropathic pain. Integration of NGS‐based mRNA and miRNA expression profiles with inverse matching and pathway analysis led to the identification of a double‐hit signature, which consisted of the upregulation of proinflammatory and proliferative genes and the repression of ECM and GAG metabolism. These findings support the important role of miRNA‒mRNA networks in defining microglial phenotypes during neuroinflammatory processes.

The present results extend and refine previous knowledge about CSF1–CSF1R signaling in neuropathic pain models. A previous study demonstrated that CSF1 released by injured neurons activates spinal microglia, inducing their proliferation and inflammatory cascades through CSF1R [7]. In line with these findings, we identified upregulated early‐response genes like Fos and Dusp2 and cytokine‐associated genes such as Cxcl2, indicating the transcriptional activation of classical inflammatory pathways. However, the present study provides additional knowledge by identifying several miRNAs, such as rno‐miR‐652‐5p, rno‐miR‐672‐5p, and rno‐miR‐455‐3p, that may serve as potential regulators of this transcriptomic reprogramming. Several miRNAs have been demonstrated to fine‐tune neuroimmune signaling, including the TNF‐α, MAPK, and IL‐17 pathways, all of which were among the significantly enriched pathways in our dataset [27–29].

An important finding of the present study was the repression of ECM‐ and GAG metabolism‐associated genes, such as Gpc6, Prelp, and Col6a2, which are critical for maintaining perineuronal nets and the glial scarring process controlling synaptic plasticity and inflammation resolution [30]. The downregulation of these genes, which may be orchestrated by miRNAs such as rno‐miR‐222‐3p, 702‐5p, 702‐3p, 664‐2‐5p, and 877, indicates an ECM‐remodeling‐prone, inflammatory microglial phenotype. Altered GAG metabolism has been linked to neuropathic pain and neurodegeneration. Recent studies have reported microglial digestion of perineuronal net–associated GAGs in neuropathic pain [31], as well as spinal cord injury [32, 33], and provided information about the broader contexts of microglial function in degeneration [34].

In addition to providing important mechanistic insight, the present study shows how integrated transcriptomic engineering can be used for identifying regulatory circuits with therapeutic potential. The two‐layer approach (NGS plus miRNA‒mRNA interaction analysis) enabled the ranking of candidate miRNAs targeting key effectors, such as Fos (Table 1), Ephb3 (Table 1), and Nr4a1 (Table 1), which participate in microglial activation and neuroimmune crosstalk and metabolic reprogramming [35, 36]. Interestingly, these studies [35, 36] suggest that NR4A family members may function as negative‐feedback regulators of microglial inflammatory responses. Therefore, the increased expression of Nr4a1 and Nr4a2 observed in the present study may reflect compensatory regulatory mechanisms activated following CSF1 stimulation rather than direct drivers of inflammation. Targeting such miRNAs, including rno‐miR‐652‐5p, rno‐miR‐672‐5p, rno‐miR‐455‐3p, and rno‐miR‐145‐5p, either as antagomirs or as miRNA mimics, may represent candidate therapeutic targets that warrant further experimental investigation.

The present work provides essential information for future studies on microglial heterogeneity and state transitions. Recent studies have reported that microglia exhibit context‐dependent transcriptomic signatures according to the injury model, anatomical region, disease stage, and even sex [37, 38]. Beyond this general diversity, recent studies have revealed dynamic state transitions over time, the emergence of spatially restricted subpopulations within the spinal cord, and concerted interactions with other glial cell types in neuropathic pain models [39–42]. These insights emphasize that microglial activation is not a uniform process but rather a spectrum of context‐driven states. The present findings suggest that miRNA regulators may serve as molecular switches for phenotypic diversification. Combining spatial transcriptomics or single‐cell RNA‐seq may elucidate their contributions to specific microglial subtypes and chronic pain.

On a larger scale, these data open new avenues for studying the epigenetic and posttranscriptional regulation of neuroinflammation. While the literature thus far has focused mainly on the role of histone acetylation and DNA methylation in glial priming [43–46], the present study complements this notion by identifying a complementary layer of posttranscriptional regulation by means of miRNA‒mRNA interaction networks. The present findings suggest that CSF1 stimulation promotes transcriptomic rewiring that involves gene induction and specific repression mediated by miRNAs targeting ECM‐associated genes, thus suggesting a dual dynamic process with a potential impact on tissue remodeling and neuronal plasticity. From a methodological point of view, the present study highlights the feasibility and power of NGS‐based systems biology approaches in neuroimmune research. Compared with conventional qPCR assays, NGS provides an unbiased, high‐throughput, and high‐resolution view of coding and noncoding RNAs. When integrated with computational miRNA–mRNA mapping, pathway enrichment analysis, and the growing body of single‐cell and spatial transcriptomic technologies [47, 48], as well as systematic NGS‐based analyses of miRNA dysregulation in neurological disease [26], this strategy offers mechanistic information that can guide functional assays and therapeutic target validation.

Several limitations of the present study should be acknowledged. First, the regulatory relationships between miRNAs and their predicted target mRNAs were inferred primarily from bioinformatics prediction and inverse expression matching. Although this integrative approach is widely used to identify candidate posttranscriptional regulatory networks, it does not establish direct causal interactions. Moreover, computational target prediction algorithms may generate both false‐positive and false‐negative interactions, and inverse expression patterns do not necessarily indicate direct miRNA‐mediated regulation because the observed transcriptional changes may arise from indirect or secondary regulatory processes. Experimental validation using luciferase reporter assays, miRNA gain‐ or loss‐of‐function experiments, or CRISPR‐based perturbation strategies will be required to confirm the direct regulatory effects of specific miRNA–mRNA pairs identified in this study. Therefore, the present work should be viewed as a systems‐level discovery framework that generates testable hypotheses regarding the role of miRNA‐mediated regulation in CSF1‐induced microglial activation. Second, the study employed primary RM cultures, which provide a controlled experimental system for isolating the direct transcriptional effects of CSF1 signaling in microglia. However, this reductionist model cannot fully reproduce the complex neuroimmune environment present in vivo, where microglia interact dynamically with neurons, astrocytes, oligodendrocytes, endothelial cells, and infiltrating immune cells. These interactions are known to influence microglial activation states and may modulate miRNA‐mediated regulatory networks during neuropathic pain. Consequently, future studies using in vivo nerve injury models and spatial or single‐cell transcriptomic approaches will be necessary to determine how the regulatory networks identified herein operate within the intact spinal neuroimmune system. Third, the present study focused on early transcriptional responses (16 h after CSF1 stimulation), which capture the initial stages of microglial activation. Microglial responses are highly dynamic and evolve over time during the transition from acute neuroinflammation to chronic pain states. Future investigations examining temporal transcriptomic changes and posttranscriptional regulatory dynamics will be important for understanding how miRNA networks contribute to different phases of neuropathic pain development. Despite these limitations, the present study provides a comprehensive transcriptomic resource identifying candidate miRNA–mRNA regulatory circuits associated with CSF1‐induced microglial activation. These findings offer a foundation for future mechanistic studies aimed at validating key regulatory interactions and exploring the therapeutic potential of miRNA‐based modulation of neuroimmune signaling. Given the recent recognition of the role of microglia in chronic pain and neurodegenerative and psychiatric disorders [5, 34, 49, 50], the studied miRNAs may be shared across disease spectra.

In the present study, we describe a miRNA‐mediated layer of regulation of CSF1‐induced microglial activation characterized by an inflammatory gene signature and ECM suppression. The data presented here provide a resource for future investigations into the role of miRNAs in regulating microglial activation. Future studies are required to determine whether modulation of specific miRNAs can alter microglial‐driven neuroinflammation while preserving physiological immune functions in the CNS. Additionally, the CSF1–CSF1R axis remains an attractive drug target. Preclinical studies have shown that CSF1R blockade reduces mechanical hypersensitivity and microgliosis in models of amyotrophic lateral sclerosis and spinal cord injury [51, 52]. In neuropathic pain models, inhibition of CSF1R signaling has also been shown to suppress microglial proliferation and mechanical hypersensitivity [7, 18]. Future studies may explore whether combining strategies targeting both ligand–receptor interactions and posttranscriptional regulators offers advantages for modulating neuroimmune responses.

## 5. Conclusion

This study provides an integrated analysis of mRNA and miRNA expression profiles in CSF1‐stimulated microglia and identifies candidate miRNA–mRNA regulatory networks associated with inflammatory signaling and ECM remodeling. The findings suggest that CSF1‐induced microglial activation is accompanied by coordinated transcriptional and posttranscriptional changes involving proinflammatory, proliferative, and ECM‐related pathways. Through the integration of NGS data, inverse miRNA–mRNA matching, and pathway enrichment analyses, several miRNAs were identified as potential regulators of genes associated with neuroinflammatory responses and ECM homeostasis.

Although the proposed miRNA–mRNA interactions remain to be experimentally validated, the present findings provide insight into the transcriptomic landscape of CSF1‐mediated microglial activation and generate testable hypotheses regarding the role of miRNA‐mediated regulation in neuroinflammation and neuropathic pain. Furthermore, the integrated transcriptomic approach employed in this study provides a valuable resource for future investigations of posttranscriptional regulatory mechanisms in microglia. Future studies incorporating functional validation and in vivo models will be important to determine the biological significance and therapeutic potential of the candidate regulatory networks identified herein.

## Author Contributions

Conceptualization: P‐H.T. and K‐C.H.; experiments: C‐C.K., R.T., and Y‐Y.L.; data analysis and presentation: P‐H.T. and K‐C.H.; statistical analyses: M‐H.H.; writing–original draft, review and editing: P‐H.T., R.T., K‐C.H., and Y‐Y.L.

## Funding

The manuscript was partly supported by grants from the Taiwan National Science and Technology Council (NSTC 112‐2314‐B‐384‐008‐MY3, NSTC 114‐2314‐B‐384‐009‐MY3).

## Disclosure

All authors have read and agreed to the published version of the manuscript.

## Ethics Statement

The experimental protocols were approved by the Animal Care Committee of Chi Mei Medical Center (reference number: 111122103, date of approval: December 21, 2022).

## Conflicts of Interest

The authors declare no conflicts of interest.

## Data Availability

The datasets generated in this study have been deposited in the Gene Expression Omnibus (GEO) under accession number GSE338938.
